# Catheter-related bloodstream *Mycobacterium wolinskyi* infection in an umbilical cord blood transplant recipient: a case report

**DOI:** 10.1186/s12879-022-07495-z

**Published:** 2022-06-04

**Authors:** Emiri Muranaka, Ryota Hase, Yoshikazu Utsu, Tomohisa Watari, Yoshihito Otsuka, Naoto Hosokawa

**Affiliations:** 1 Department of Infectious Diseases, Japanese Red Cross Narita Hospital, 90-1 Iidacho, Narita, Chiba Japan; 2 Department of Infectious Diseases, Kameda Medical Center, Kamogawa, Chiba Japan; 3 Department of Hematology and Oncology, Japanese Red Cross Narita Hospital, Narita, Chiba Japan; 4 Department of Clinical Laboratory, Kameda Medical Center, Kamogawa, Chiba Japan

**Keywords:** *Mycobacterium wolinskyi*, Rapidly growing mycobacteria, Umbilical cord blood transplant, Unidentifiable Gram-positive rods, Case report

## Abstract

**Background:**

Catheter-related bloodstream infection (CRBSI), caused by rapidly growing mycobacteria (RGM), is a rare infectious complication in hematopoietic stem cell transplant (HSCT) recipients and can often be misdiagnosed as Gram-positive rod (GPR) bacteremia.

**Case presentation:**

We present a case of CRBSI caused by *Mycobacterium wolinskyi*, a rare RGM, in a 44-year-old female patient who received an umbilical cord blood transplant.

**Conclusions:**

Rapidly growing mycobacteria can stain as GPRs and may grow in routine blood culture media after 3–4 days of incubation. These features are not widely known to clinicians, and acid-fast staining is therefore recommended when unidentifiable GPRs are detected in blood cultures, especially in immunocompromised patients, such as those with hematologic malignancies or intravascular devices.

## Background

*Mycobacterium wolinskyi* is a rapidly growing non-tuberculous mycobacteria (NTM) that belongs to the *Mycobacterium smegmatis* group [1]. Infections caused by *M. wolinskyi* are rare, with less than 30 cases reported to date. Most of them are skin and soft-tissue infections or prosthetic joint infections after trauma or surgery, and only four were bloodstream infections [2–5].

In hematopoietic stem cell transplant (HSCT) recipients, catheter-related bloodstream infections (CRBSI) are the most commonly encountered NTM infectious complications [6, 7], mostly caused by rapidly growing mycobacteria (RGM) [7], which are defined as mycobacteria growing within 7 days [8]. They may grow in routine blood culture media after 3–4 days of incubation [8, 9]. However, the diagnosis is often difficult and delayed, since RGM may be misidentified as Gram-positive rods (GPRs) rather than acid-fast bacilli [10–14].

Here, we have described a case of peripherally inserted central catheter-associated bloodstream infection due to *M. wolinskyi*, diagnosed by acid-fast staining in a second umbilical cord blood transplant recipient. We have also reviewed the clinical course and outcomes of previously reported *M. wolinskyi* bacteremia.

## Case presentation

A 44-year-old woman had undergone first allogeneic cord blood transplantation (CBT, 2.35 × 10^6^/kg nucleated cells, two locus mismatch) for acute myeloid leukemia after two courses of induction therapy that led to hematologic complete remission. The patient was pre-treated with cytarabine, cyclophosphamide, and total body irradiation conditioning. Graft-versus-host disease prophylaxis consisted of cyclosporine and methotrexate. Her medical, family, and social histories were unremarkable. The patient underwent a second CBT (2.0 × 10^7^/kg nucleated cells) 32 days after the first due to graft failure.

**Fig. 1:**
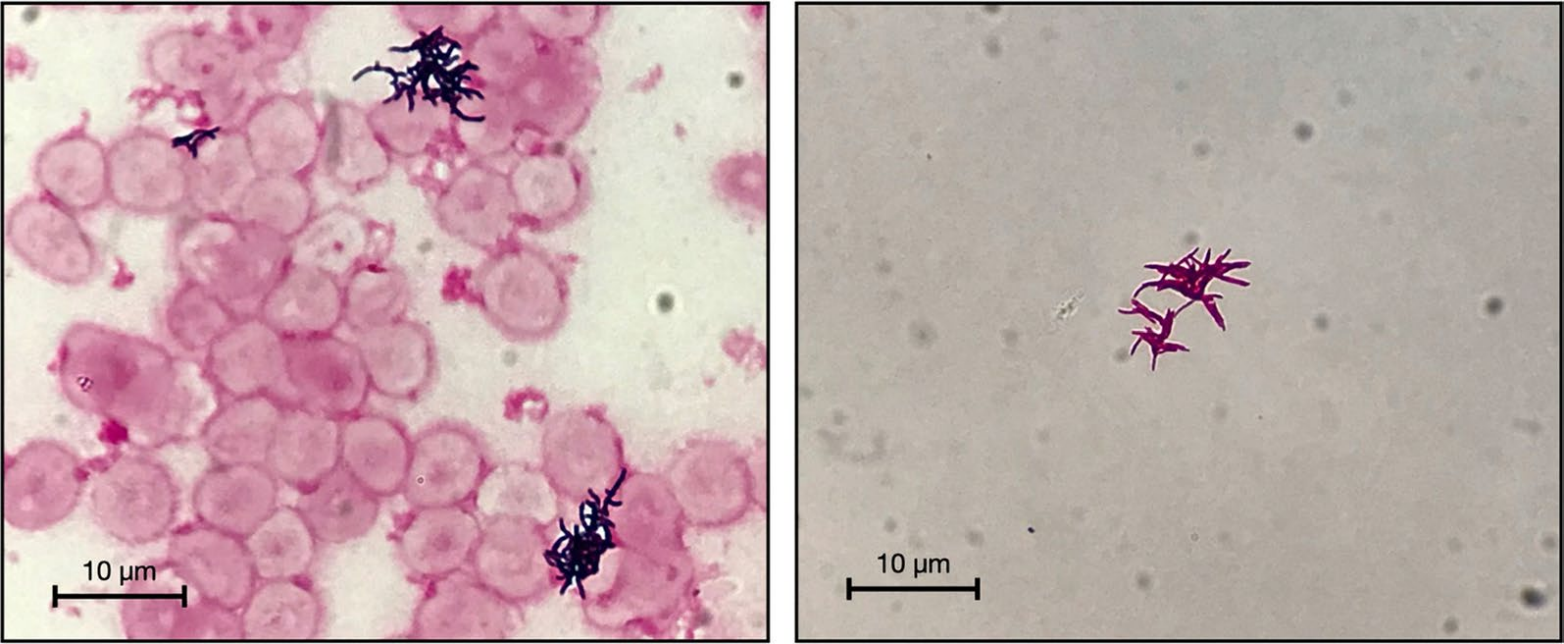
*Left* Gram staining of blood cultures shows diphtheroid bacilli and short branching filaments. *Right* Ziehl–Neelsen staining of colonies obtained by subculture

Seven days after the second CBT, she presented with high fever and shaking chills. Upon physical examination, painful induration with linear erythema along the superficial veins was observed in the peripherally inserted central catheter (PICC) site, which was inserted on the day after the second CBT. Two sets of blood samples were drawn for culture and meropenem treatment was started. The blood culture was positive on the third day of incubation (10 days after the second CBT). Gram staining of a positive blood culture revealed GPR (Fig. 1, left), and vancomycin was added thereafter. Fever persisted and PICC was removed on day 11 after the second CBT (PICC was maintained for 10 days). The VITEK® 2 system (bioMérieux, Durham, NC, USA) was not able to identify the organism. Blood cultures drawn on days 10, 11, 15, and 22 after CBT were also positive for GPR. On the 14th day after the second CBT, Ziehl–Neelsen staining was performed and was found to be positive for acid-fast bacilli (Fig. 1, right).

The nucleotide sequences were analyzed using the National Center for Biotechnology Information BLAST (http://blast.ncbi.nlm.nih.gov). The almost full-length (1442-bp) 16S rDNA gene sequence of the isolate shared 100% similarity to *M. wolinskyi* type strain ATCC 700010.

In addition to PICC removal, imipenem/cilastatin (IPM/CS), amikacin (AMK), levofloxacin (LVX), and azithromycin (AZM) were started empirically. Antibiotic susceptibilities were determined using the broth microdilution method (BrothMIC RGM®; Kyokuto, Tokyo, Japan) based on the Clinical and Laboratory Standards Institute M24 recommendations (Table 1) [15].

**Table 1:** Results of antimicrobial susceptibility testing of *Mycobacterium wolinskyi* isolated in our case

Antibiotic	MIC (mg/mL)	S ≤	I	R >
Amikacin	≤ 4	16	32	64
Tobramycin	> 16	4	8–16	32
Imipenem	16	4	8–16	32
Levofloxacin	2	2	4	8
Moxifloxacin	1	1	2	4
Clarithromycin	64	2	4	8
Trimethoprim–sulfamethoxazole	152/8	38/2	No	76/4
Doxycycline	≤ 0.5	1	2–8	16
Linezolid	2	8	16	32

*MIC* minimum inhibitory concentration, *S* susceptible, *I* intermediate, *R* resistant

According to the susceptibilities, IPM/CS and AZM were discontinued, and minocycline (MIN) was added. LVX was changed to moxifloxacin (MFX) based on previous case series [4]. After 1 month of intravenous administration of AMK, the patient was discharged on an oral regimen of MFX and MIN. Although MXF had to be discontinued after 4 months due to nausea, MIN was continued for 6 months.

After 3 weeks of combination antimicrobial therapy, blood cultures became negative. She showed successful engraftment by day 28. The patient responded well to therapy and no recurrence of infection was identified at 1-year follow-up.

## Discussion and conclusion

Herein, we have described a case of PICC-related bloodstream *M. wolinskyi* infection in an umbilical cord blood transplant recipient. The findings suggested that acid-fast staining should be conducted when unidentifiable GPRs are detected in blood cultures, especially in immunocompromised patients with long-term indwelling catheters.

Misidentification of RGM as GPRs, including *Corynebacterium* spp. [11, 14, 16, 17], *Rhodococcus*, *Brevibacterium* [10], *Actinomyces*, or *Nocardia* [17, 18], had been reported earlier and performing acid-fast staining is recommended for cases in which Gram-positive bacilli have been cultured from high-risk patients. One study at a quality-control center in Switzerland [13], in which investigators delivered *M. fortuitum* specimens labelled as ‘pus from an abscess’ to 50 laboratory facilities, had shown only 13 of 50 (26%) to be correctly identified as “RGM” or “*M. fortuitum*”; 46% were incorrectly identified as *Nocardia* sp., 8% as *Rhodococcus* sp. Gram-positive rods, *Actinomyces*, *Streptococcus*, or *Corynebacterium*. Notably, all laboratories that did not use acid-fast stains were unable to correctly identify the organisms.

We reviewed all available literature for the five cases of *M. wolinskyi* bloodstream infections, including the present one (Table 2). Three of the five cases had hematologic malignancies as comorbidities. In all three cases for which Gram stain results were available, blood cultures showed GPRs. An intravascular device was present in 4 of the 5 cases, and the device was removed in 3 cases. Blood cultures were positive within 2 to 5 days of incubation for all cases. The prognosis was generally good, especially for those whose devices had been removed. No death was reported during the treatment.

**Table 2:** Summary of published cases of *M. wolinskyi* bacteremia

	Age	Gender	Comorbidities	Type of infection	Treatment	Intravascular devices	Device removal	Gram stain	Outcome	Time to positive blood culture (days)
Chen et al. [2]	22	f	NHL on chemotherapy	BSI/septic arthritis in native joint	Surgical debridement + AMK (1 month), MXF and MIN (6 months)	Venous port	Yes	N/A	N/A	3
Ohno et al. [3]	55	f	CML on chemotherapy	BSI No source identified	AMK (1 month), MIN and LVX (6 months)	None	N/A	GPR	Good outcome at 1.5-year follow-up	5
Ariza-Heredia et al. [4]	16	m	Congenital aortic stenosis status post-Ross procedure	BSI, likely endocarditis and infected aortic root graft	AMK, MXF and DOXY (ongoing)	Vascular graft	Yes	N/A	Underwent pulmonary artery conduit replacement	5
Kitajima et al. [5]	82	m	Status-post AVR and MVR	Prosthetic valve endocarditis	AMK, IPM and CLR (6 weeks) then CIP and MIN (total 12 months)	Prosthetic valve	No	GPR	Cure	5
Present case	43	f	AML post hematopoietic stem cell transplantation	Catheter related blood stream infection	AMK, IPM/CS, LVX and AZM (6 days) AMK, IPM/CS, LVX and MIN (13 days), AMK, MXF and MIN (15 days) then MXF (4 months) and MIN (6 months)	PICC	Yes	GPR	Cure	2

*NHL* non-Hodgkin lymphoma, *CML* chronic myeloid leukemia, *AVR* aortic valve replacement, *MVR* mitral valve replacement, *AML* acute myeloid leukemia, *BSI* bloodstream infection, *AMK* amikacin, *MXF* moxifloxacin, *MIN* minocycline, *LVX* levofloxacin, *IPM* imipenem, *CLR* clarithromycin, *CIP* ciprofloxacin, *AZM* azithromycin, *PICC* peripherally inserted central catheter, *IPM/CS* imipenem/cilastatin, *N/A* not available, *GPR* Gram-positive rod

We reported a case of peripherally inserted central catheter-associated bloodstream infection caused by *Mycobacterium wolinskyi* in a second umbilical cord blood transplant recipient. *M. wolinskyi* is an RGM and a rare cause of bacteremia in immunosuppressed patients with hematologic malignancies or intravascular devices.

Rapidly growing mycobacteria may grow in routine blood culture media and sometimes be confused with Gram-positive rods, resulting in delayed diagnosis. In immunocompromised patients or those with intravascular devices and bacteremia caused by “unidentifiable Gram-positive rods”, acid-fast staining should be performed.

## Data Availability

Data sharing is not applicable to this article as no datasets were generated or analyzed during the current study.

## Abbreviations

CRBSI: Catheter-related bloodstream infection
RGM: Rapidly growing mycobacteria
HSCT: Hematopoietic stem cell transplant
GPR: Gram-positive rod
NTM: Non-tuberculous mycobacteria
CBT: Cord blood transplantation
PICC: Peripherally inserted central catheter
IPM/CS: Imipenem/cilastatin
AMK: Amikacin
LVX: Levofloxacin
AZM: Azithromycin
MXF: Moxifloxacin
MIN: Minocycline
